# Why Not?

**Published:** 1866-09

**Authors:** W. R. Storer

**Affiliations:** Boston


### WHY NOT?

### A BOOK FOR EVERY WOMAN.

The Prize Essay to which the American Medical Society awarded the Gold Medal for 1865.

By W. R. Storer, M. D., Boston.

This is a most excellent little work—well written. The subject upon which it treats is one of momentous importance to almost every woman; but most emphatically so to those guilty of the practices which it condemns.

The subject is handled in a most masterly manner, yet it is very apparent that the author used but a tithe of the material at his command, and doubtless the greater labor was to select rather than to find material for the work. It should be in the hands of every woman, that those guilty of the practices it condemns may reform, and that others may be warned never to enter such a course. Many, of course, entertain correct views upon this subject, who could not be induced to practice the vice here condemned. For such persons the work is not intended, except so far as it may aid them in correcting the evil in others.

The work should bé published in the cheapest substantial form, and effort made for its largest possible distribution. This we understand to be the aim of the Association.

It is now put up in two forms, one a binding in fine cloth at $1, and in paper at 50 cents. It is for sale by Robert Clark & Co., Cincinnati.

